# Supramolecular chirality in self-organised systems and thin films

**DOI:** 10.1039/d4na90108h

**Published:** 2024-10-28

**Authors:** G. Giancane, S. Bettini, L. Valli

**Affiliations:** a Department of Cultural Heritage, University of Salento Via D. Birago 64 Lecce 73100 Italy; b Department of Biological and Environmental Sciences and Technologies (DiSTeBA), University of Salento, Campus Ecotekne Via per Monteroni Lecce 73100 Italy

## Abstract

Gabriele Giancane, Simona Bettini and Ludovico Valli introduce the *Nanoscale Advances* themed collection on Supramolecular chirality in self-organised systems and thin films.
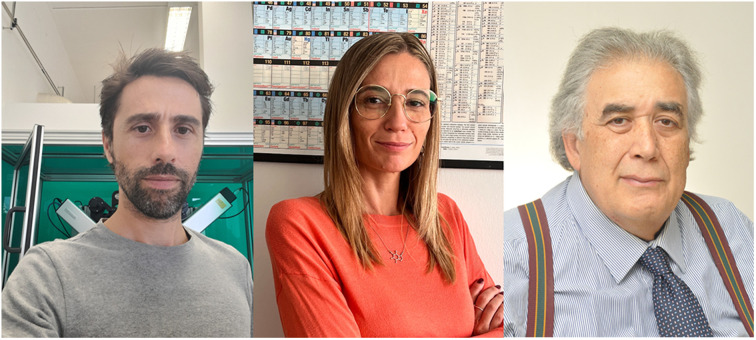

In the fascinating world of molecular science, chirality holds a special place by reason of its key role in asymmetry, dictating the behavior and interactions of molecules in nature. From the twisted helix of DNA to the way light interacts with certain compounds, chirality is everywhere. At its most basic level, chirality describes a property of an object that cannot be superimposed on its mirror image, much like our left and right hands. But when chirality extends beyond individual molecules to larger, more complex assemblies, we enter the intriguing realm of supramolecular chirality.

Supramolecular chirality is the manifestation of chirality in assemblies of molecules, driven by non-covalent interactions such as hydrogen bonding, van der Waals forces, or π–π stacking with other molecules or special surfaces (https://doi.org/10.1039/D3NA00894K). What makes supramolecular chirality particularly captivating is that it emerges not from the individual building blocks themselves, but from the collective organization of these molecules. This phenomenon can lead to the formation of chiral structures, such as helices or twisted ribbons, even when the constituent molecules themselves are achiral (https://doi.org/10.1039/D1NA00531F).

One compelling example of supramolecular chirality can be found in liquid crystals, where the self-organization of molecules into twisted structures (https://doi.org/10.1039/D4NA00353E) gives rise to fascinating optical properties (https://doi.org/10.1039/D0NA01070G). Similarly, in biological systems, supramolecular chirality is fundamental to the functioning of many macromolecular assemblies, such as the chiral arrangement of proteins and lipids in cell membranes, which plays a key role in cell signaling and function.

A particularly exciting application of supramolecular chirality is in the field of chiral sensing (https://doi.org/10.1039/D0NA00127A). Chiral sensors are designed to detect enantiomers by exploiting the unique interactions between chiral analytes and chiral sensor molecules and/or aggregates. Supramolecular assemblies can amplify these subtle differences in chirality, making it possible to achieve highly selective detection. This has profound implications for fields like pharmacology, where the ability to distinguish between enantiomers is critical since the different mirror images of a drug can have drastically different biological effects.

Supramolecular chiral sensors are also proving valuable in environmental monitoring, where they can be used to detect pollutants, toxins, or even drugs with high specificity. By incorporating chirality into the design of these sensors, researchers can create systems that respond selectively to chiral molecules in complex environments (https://doi.org/10.1039/D4NA00217B). This not only enhances sensitivity, but also offers opportunities for real-time monitoring and diagnostics.

Beyond biology and sensing, supramolecular chirality is also at the forefront of cutting-edge materials science (https://doi.org/10.1039/D4NA00027G). Researchers are harnessing this property to create new materials with unique functionalities (https://doi.org/10.1039/C8NA00159F), such as chiral catalysts and even potential applications in nanotechnology (https://doi.org/10.1039/D3NA00301A, https://doi.org/10.1039/D3NA00192J). These supramolecular systems offer promising pathways for innovation (https://doi.org/10.1039/D3NA00808H), as they allow scientists to design materials that mimic the complexity and precision of biological systems.

In the broader scope of chemistry, chirality has always been a fundamental concept, but supramolecular chirality introduces an additional layer of complexity and opportunity (https://doi.org/10.1039/D2NA00789D). By understanding and controlling how molecules organize themselves into chiral structures (https://doi.org/10.1039/C8NA00216A), we are not only unveiling new scientific insights but also unlocking powerful tools for technological advancement (https://doi.org/10.1039/D1NA00425E, https://doi.org/10.1039/D1NA00284H).

As we continue to explore supramolecular chirality, especially in fields like sensing and materials science, it's clear that this concept will play a pivotal role in shaping innovations across disciplines. Whether in medicine, environmental science, or technology, supramolecular chirality is poised to make a significant impact on how we detect, understand, and manipulate the molecular world around us.

As Guest Editors of this themed collection, we would like to express our sincere appreciation to the authors for their high-quality contributions and to the referees for their diligent work, which ensured the excellence of this collection. Finally, we extend our gratitude to the Editorial Staff of *Nanoscale Advances* for their dedicated efforts and ongoing support.

